# Data on the effect of sex on the size, cellular content, and neuronal density of the developing brain in mice exposed to isoflurane and carbon monoxide

**DOI:** 10.1016/j.dib.2017.06.028

**Published:** 2017-06-17

**Authors:** Li Wang, Aili Wang, William W. Supplee, Kayla Koffler, Ying Cheng, Zenaide M.N. Quezado, Richard J. Levy

**Affiliations:** a The Sheikh Zayed Institute for Pediatric Surgical Innovation, Division of Pain Medicine, Children׳s National Health System, Children׳s Research Institute, The George Washington University School of Medicine and Health Sciences, United States; b Department of Anesthesiology, Columbia University Medical Center, United States; c Rutgers New Jersey Medical School, United States; d Center for Genetic Medicine Research, Children׳s National Health System, Children׳s Research Institute, The George Washington University School of Medicine and Health Sciences, United States

## Abstract

The data presented here detail the changes in size, cellular content, and neuronal density of the developing brain over time with respect to sex in C57Bl/6 mice following neonatal exposure to isoflurane, carbon monoxide, or their combination. Specifically, brain weight- and brain volume-to-body weight ratios are presented, representative immunoblots of whole brain cell-specific protein content are depicted, and quantification of the number of neurons in the primary somatosensory cortex and CA3 region of the hippocampus are shown. Three discrete postnatal time points are represented: P7 (prior to exposure), P14 (one-week post exposure), and P42-56 (5–7 weeks post exposure). Major findings from the data presented here are reported in the manuscript “Carbon Monoxide Incompletely Prevents Isoflurane-induced Defects in Murine Neurodevelopment" (Wang et al., in press) [1].

**Specifications Table**

**Table:** 

Subject area	*Biology*
More specific subject area	*Developmental neuroscience*
Type of data	*Figures*
How data was acquired	*Analytical balance, microscope, densitometry*
Data format	*Analyzed*
Experimental factors	1-h exposure to 0 ppm (air), 5 ppm, or 100 ppm CO in air with or without isoflurane (2%) on postnatal day 7
Experimental features	Brain size, whole brain cellular content, and neuronal density in primary somatosensory cortex and hippocampal CA3 region were measured 1-week or 5–7 weeks post exposure along with 7-day old, unexposed controls. Brain and body weight were measured with an analytical balance. Brain volume was determined from the amount of saline displaced by fresh whole brain. Immunoblot was performed followed by chemiluminescence and densitometry. Neuronal density was determined from slide mounted cresyl violet stained brain sections.
Data source location	*New York, New York, USA, and Washington, D.C., USA*
Data accessibility	The data are with this article

**Value of the data**

• The data assess for age- and sex-based changes within the developing brain in a postnatal model of anesthesia-induced neurotoxicity.
• The findings provide insight into the independent effects of age and sex at multiple time points following neonatal exposure.
• The results will help researchers with experimental design in future investigation. Specifically, the data will serve as a benchmark for inclusion of both sexes in experimental cohorts.

## 1 Data

**Fig. 1:**
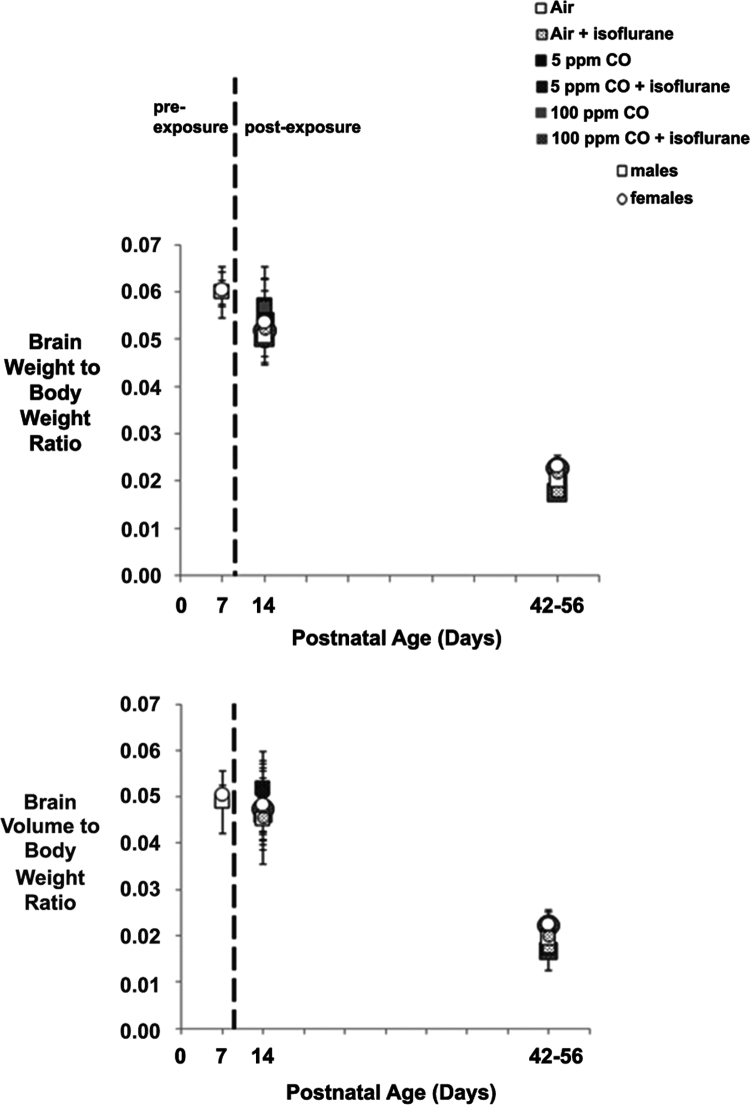
Normalized brain size following exposure with respect to sex. Brain weight-to-body weight and brain volume-to-body weight ratios are depicted for each cohort 1-week (postnatal day 14) and 5–7 weeks (postnatal days 42–56) post exposure. Values for unexposed, naïve controls on postnatal day 7 are also shown. Males are indicated with a square marker and females are indicated with a circle. Dotted line separates pre- and post-exposed cohorts. *N* = 10 animals for each exposed cohort (5 males and 5 females). *N* = 6 animals for unexposed controls (3 males and 3 females). Values are expressed as means ± standard deviation. There was no significant difference between cohorts based on sex.

**Fig. 2:**
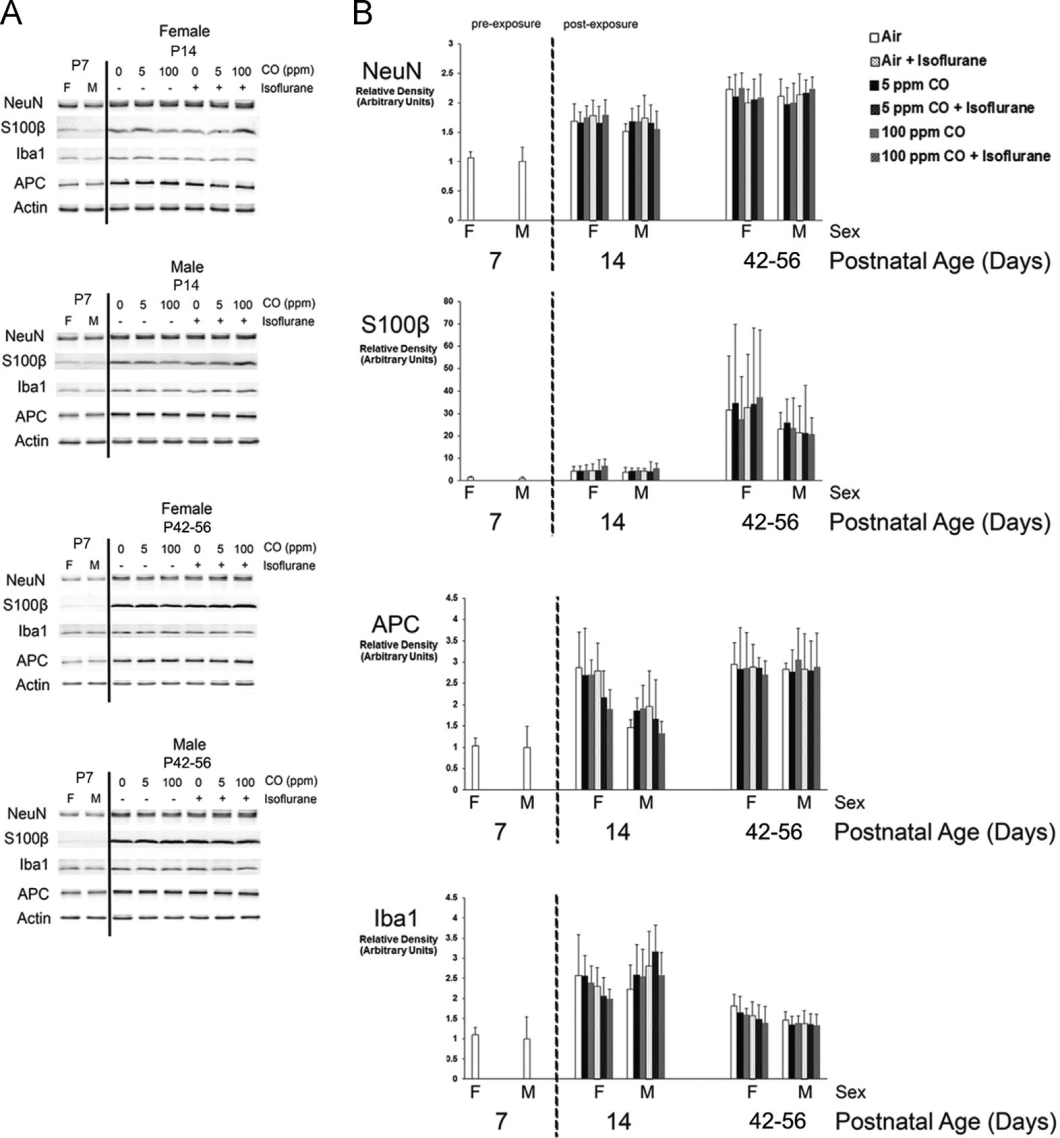
Cell-specific protein content in whole brain following exposure with respect to sex. Steady-state levels of neuron-, astrocyte-, oligodendrocyte-, and microglial-specific protein were quantified in whole brain 1-week (postnatal day 14) or 5–7 weeks (postnatal days 42–56) post exposure. Seven-day old naïve female #3 and male #1 mice were arbitrarily chosen as representative unexposed controls to permit age- and sex-based comparison. A. Representative immunoblots of neuron specific antigen (NeuN), S100β, adenomatous polyposis coli (APC), and ionized calcium binding adapter molecule 1 (Iba1) are depicted for female and male mice on postnatal day 14 (P14) and postnatal days 42–56 (P42-56). Actin was used as a loading control. Exposure cohorts are indicated by concentration of carbon monoxide (CO) (0 ppm (ppm) [air], 5 ppm, or 100 ppm) with (+) or without (−) isoflurane. P7 female (F) and male (M) controls are indicated. B. Graphical representation of relative densities of NeuN, S100β, APC, and Iba1 based on sex are shown. Sex of the cohort is indicated (F or M). Dotted line separates pre- and post-exposed cohorts. Values are expressed as means plus standard deviation. Unexposed, naïve P7 male values were arbitrarily set to 1. *N* = 10 animals (5 males and 5 females) for each exposed cohort per time point. There was no significant difference between cohorts based on sex.

**Fig. 3:**
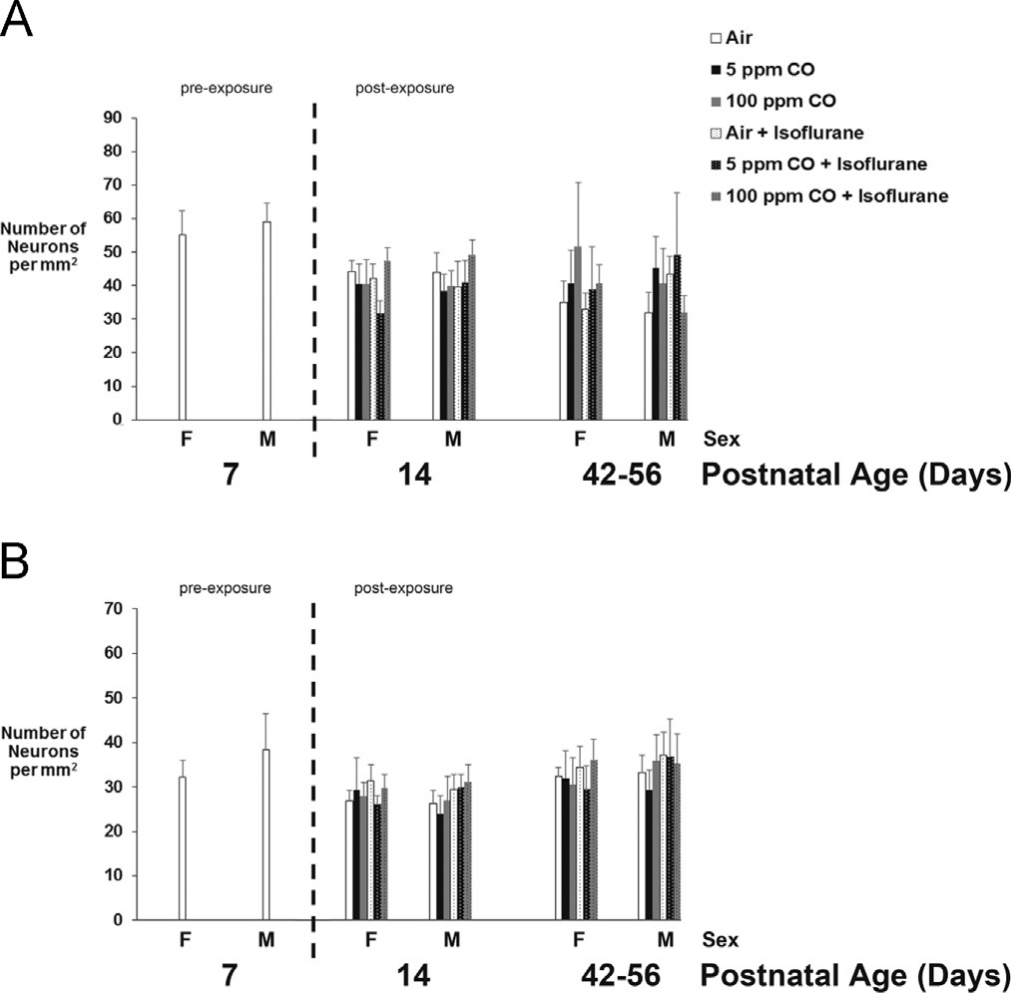
Estimated neuron density in primary somatosensory cortex and hippocampus following exposure with respect to sex. Quantification of the number of neurons (in thousands) in A. primary somatosensory cortex and B. CA3 region of the hippocampus for each cohort 1-week (postnatal day 14) and 5–7 weeks (postnatal days 42–56) post exposure as well as for unexposed, naïve controls on postnatal day 7 is demonstrated. Dotted line separates pre- and post-exposed cohorts. Sex of the cohort is indicated (F or M). Values are expressed and means plus standard deviation. *N* = 4 animals per exposure cohort per time point. There was no significant difference in number of neurons in primary somatosensory cortex or CA3 region between cohorts based on sex.

The data presented here are values obtained from developing C57Bl/6 mice on P7 (prior to exposure), P14 (1-week post exposure), and P42-56 (5–7 weeks post exposure) with respect to sex. Specifically, brain weight- and brain volume-to-body weight ratios are demonstrated, steady-state levels of whole brain cell-specific protein content are shown, and quantification of neuronal density in the primary somatosensory neocortex and CA3 region of the hippocampus is presented (Fig. 1, Fig. 2, Fig. 3 and Table 1, Table 2, Table 3).

## 2 Experimental design, materials and methods

### 2.1 Animal exposures

The care of the animals in this study was in accordance with NIH and Institutional Animal Care and Use Committee guidelines. Study approval was granted by the Children׳s National Medical Center and Columbia University Medical Center. Six to eight week old breeding pairs of C57Bl/6 mice (20–30 g) were acquired (Charles River, Wilmington MA) to yield newborn pups. On P7, we exposed male and female C57Bl/6 mouse pups to air (0 ppm CO), 5 ppm CO in air, or 100 ppm CO in air with and without isoflurane (2 vol%) for 1 h in a 7-liter Plexiglas chamber (25 cm×20 cm×14 cm) as previously described [1], [2]. An equal number of male and female mice were evaluated and were randomly exposed to a designated CO concentration with or without isoflurane. Following exposure, pups were returned to their respective dams. P7 was chosen because synaptogenesis peaks at day 7 in rodents and is completed by the second or third week of life [3], [4]. One-hour exposure to 2% isoflurane has previously been shown to induce neuronal degeneration in 7 day old mice and represents a brief anesthetic exposure [2], [5]. Furthermore, 1-h exposure to such concentrations of CO inhibited isoflurane-induced apoptosis in various regions of mouse forebrain in a dose-dependent manner [2].

### 2.2 Brain weight and volume

Separate cohorts of mice were evaluated 1-week post exposure or 5–7 weeks post exposure. An unexposed, naïve cohort was also evaluated on P7. Following euthanasia with pentobarbital (150 mg/kg, ip), body weight, brain weight, and brain volume were determined as previously described [1]. Brain weight-to-body weight ratios and brain volume-to-body weight ratios were then calculated.

### 2.3 Immunoblot analysis

10 µg samples of homogenized whole brain protein were subjected to SDS-acrylamide gel electrophoresis and immunoblotting as previously described [1].

### 2.4 Estimate of neuronal density

Cresyl violet-positive neurons with a clear nucleus and nucleoli in the primary somatosensory cortex and in the pyramidal layer of the CA3 region of the hippocampus were counted by a blinded observer (K.K.) as previously described [1].

### 2.5 Statistical analysis

Sample size was determined based on the number of mice required to detect a 20% change in NeuN content. A sample size of 8 animals per cohort provided a power of 80 based on an α of .05. Mice were randomly assessed either 1-week post exposure (P14) or 5 to 7 weeks post exposure (P42-56). Equal number of males and females were assessed for each experiment. Ten animals per exposure cohort per time point were utilized for measurement of brain size and immunoblot analysis (providing a power of 90), while 4 animals per cohort per time point were used for slide mounted sections (providing a power of 80). A cohort of 10 (equal number of males and females) healthy, unexposed 7 day old mice were also assessed as naïve controls. Six of these mice were utilized for brain size assessment and immunoblot analysis while 4 were used for slide mounted sections.

Data was assessed for normality by examining histograms and box plots. Parametric or non-parametric tests were applied based on assumptions of normal distribution or failure to meet requirements. Statistical significance was assessed using two-way ANOVA and post hoc Tukey׳s test for brain size, and cell-specific protein densities. Regression models were utilized to determine significance of sex for each outcome measure. Unpaired two-tailed Student׳s *t*-test was used to assess cell-specific protein densities between sexes in 7-day old, unexposed naïve controls. Kruskal–Wallis test with Dunn׳s multiple comparison post hoc test was utilized for neuronal density values. Data are presented as mean with standard deviation and significance was set at *P* < .05.

**Table 1:** Normalized brain size following exposure with respect to sex.

**7 d old**	**Sex**	**Brain wt/body wt**	**Brain Volume/body wt**	
	**Female**	**0.0605**	**0.0505**	(mean)
		0.0037	0.0020	(st dev)
	**Male**	**0.0599**	**0.0488**	(mean)
		0.0025	0.0068	(st dev)

Mean values with standard deviation are shown for Fig. 1. Brain weight-to-body weight and brain volume-to-body weight ratios are provided for each cohort 1-week (postnatal day 14) and 5–7 weeks (postnatal days 42–56) post exposure. Values for unexposed, naïve controls on postnatal day 7 are also listed.

**Table 2:** Normalized brain size following exposure with respect to sex.

**14 d**	**Sex**	**Brain wt/body wt**	**Brain volume/body wt**	
**Air**	**Female**	**0.0537**	**0.0482**	(mean)
		0.0046	0.0059	(st dev)
	**Male**	**0.0504**	**0.0453**	(mean)
		0.0054	0.0068	(st dev)
**5** **ppm**	**Female**	**0.0514**	**0.0455**	(mean)
		0.0089	0.0101	(st dev)
	**Male**	**0.0541**	**0.0479**	(mean)
		0.0087	0.0083	(st dev)
**100** **ppm**	**Female**	**0.0527**	**0.0482**	(mean)
		0.0025	0.0026	(st dev)
	**Male**	**0.0567**	**0.0516**	(mean)
		0.0059	0.0061	(st dev)
**Air/iso**	**Female**	**0.0504**	**0.0441**	(mean)
		0.0060	0.0045	(st dev)
	**Male**	**0.0520**	**0.0461**	(mean)
		0.0038	0.0053	(st dev)
**5** **ppm/iso**	**Female**	**0.0569**	**0.0509**	(mean)
		0.0084	0.0089	(st dev)
	**Male**	**0.0572**	**0.0518**	(mean)
		0.0054	0.0054	(st dev)
**100** **ppm/iso**	**Female**	**0.0519**	**0.0473**	(mean)
		0.0055	0.0055	(st dev)
	**Male**	**0.0502**	**0.0462**	(mean)
		0.0057	0.0057	(st dev)

Mean values with standard deviation are shown for Fig. 1. Brain weight-to-body weight and brain volume-to-body weight ratios are provided for each cohort 1-week (postnatal day 14) and 5–7 weeks (postnatal days 42–56) post exposure. Values for unexposed, naïve controls on postnatal day 7 are also listed.

**Table 3:** Normalized brain size following exposure with respect to sex.

**42–56 d**	**Sex**	**Brain wt/body wt**	**Brain volume/body wt**	
**Air**	**Female**	**0.0231**	**0.0224**	(mean)
		0.0013	0.0027	(st dev)
	**Male**	**0.0200**	**0.0195**	(mean)
		0.0010	0.0019	(st dev)
**5** **ppm**	**Female**	**0.0233**	**0.0207**	(mean)
		0.0020	0.0026	(st dev)
	**Male**	**0.0204**	**0.0203**	(mean)
		0.0029	0.0029	(st dev)
**100** **ppm**	**Female**	**0.0219**	**0.0212**	(mean)
		0.0019	0.0039	(st dev)
	**Male**	**0.0187**	**0.0166**	(mean)
		0.0031	0.0041	(st dev)
**Air/iso**	**Female**	**0.0220**	**0.0201**	(mean)
		0.0013	0.0013	(st dev)
	**Male**	**0.0179**	**0.0176**	(mean)
		0.0005	0.0011	(st dev)
**5** **ppm/iso**	**Female**	**0.0227**	**0.0209**	(mean)
		0.0008	0.0015	(st dev)
	**Male**	**0.0189**	**0.0172**	(mean)
		0.0017	0.0015	(st dev)
**100** **ppm/iso**	**Female**	**0.0227**	**0.0222**	(mean)
		0.0010	0.0034	(st dev)
	**Male**	**0.0175**	**0.0167**	(mean)
		0.0013	0.0018	(st dev)

Mean values with standard deviation are shown for Fig. 1. Brain weight-to-body weight and brain volume-to-body weight ratios are provided for each cohort 1-week (postnatal day 14) and 5–7 weeks (postnatal days 42–56) post exposure. Values for unexposed, naïve controls on postnatal day 7 are also listed.
